# Commentary: Efficacy and safety of low-molecular-weight collagen peptides in knee osteoarthritis: a randomized, double-blind, placebo-controlled trial

**DOI:** 10.3389/fnut.2025.1712455

**Published:** 2025-11-20

**Authors:** Junpeng Qiu, Fangjun Xiao, Junxing Yang

**Affiliations:** 1 Shenzhen Hospital (Futian) of Guangzhou University of Chinese Medicine, Shenzhen, China; 2 The Sixth Clinical Medical College, Guangzhou University of Chinese Medicine, Guangzhou, China

**Keywords:** knee osteoarthritis, low-molecular-weight collagen peptides, methodological critique, clinical significance, evidence-based medicine

The randomized controlled trial by Park et al. (1) adds to a growing body of research on nutritional interventions for knee osteoarthritis (OA). The authors conclude that daily supplementation with 3,000 mg of low-molecular-weight collagen peptides (LMCP) over 6 months led to significant improvements in WOMAC pain and function scores among patients with early-stage disease. While these findings add to current interest in nutraceutical approaches for OA, their value in practice largely rests on how rigorous the study design was, whether the outcomes are clinically meaningful, and whether the proposed mechanism makes biological sense.

There are aspects of the study design which raise concern about the internal validity of the outcomes. Successful randomization is essential to the internal validity of any RCT, as it ensures baseline comparability between treatment groups. Park et al. noted a baseline imbalance in Kellgren–Lawrence grades (*p* = 0.037), with the placebo group including a much larger proportion of grade I patients (77.4%) than the LMCP group (51.7%). Imbalances of that sort can occur with the absence of a complete randomization procedure, and lead to residual confounding (2). Since the LMCP group had more advanced disease at baseline, they might have shown greater improvement regardless of treatment. This apparent prognostic factor was not addressed by the authors, for instance by the application of analysis of covariance, thus rendering their claims about efficacy of LMCP less convincing. Moreover, the study had a 25% attrition rate. Although the Last Observation Carried Forward method was used for this, a high drop-out rate is worrying and less convincing if it follows that the reasons for withdrawal may be related to response to treatment or side effects. The per protocol analysis used, as opposed to intention to treat, may have over-estimated the apparent degree of treatment effect. Also it does not represent how the intervention would have acted in the real life situation (3). Thus, taken as a whole, the effect of the randomization imbalance and the considerable drop-out leads to a questioning of the internal validity of this study.

In addition to methodological problems, the interpretation of the statistical power and results of the study should also be critically examined. Sample size determination was done on the WOMAC total, rather than the primary outcome being the WOMAC pain that had been defined previously to be the WOMAC pain score. This inconsistency makes more difficult to interpret the study's findings. After the application of attrition, the study may not have had enough power to show a significant effect on its own primary endpoint. Another point of note is the discrepancy between measures of pain: WOMAC pain was significantly different between groups whereas this was not the case for the Visual Analog Scale (*p* = 0.299). This result would indicate that the effect of the treatment might differ by the instrument used for measurement, thereby calling into question the reliability of the results achieved with respect to pain.

In examining the results, it is important to consider both the clinical significance of the results and the plausibility of the suggested mechanism. Statistical significance does not equate clinical significance. The LMCP group showed a mean reduction of 1.9 points on the WOMAC pain subscale (0–20). In order to provide some context for this result we considered established thresholds for the minimal clinically important difference (MCID). The standardized mean difference reported for the comparison (SMD) in the study was approximately 0.46 which could be compared with an SMD based MCID of about 0.39 which is frequently cited in orthopedic literature (4). More informatively, when compared with absolute MCID estimates based on anchors which were synthesized in a recent systematic investigation to be in the range of about 7–21 points on the 0–100 WOMAC pain scale (1.4–4.2 points on the 0–20 point scale used in this trial), the mean observed change of −1.9 occurred in the vicinity of the lower limit of these estimates (5). The absolute change was modest and associated with a large standard deviation (±4.14), suggesting inconsistent responses among participants. It is not reported how many patients met the OMERACT-OARSI responder criteria which is a more patient-centered and clinically relevant outcome (6, 7). Without such data it is difficult to assess how many participants had a significant improvement. In addiction, the authors suggest that LMCP may increase type II collagen synthesis and protect cartilage matrix. However, joint space width and inflammatory markers such as ESR and hs-CRP showed no intergroup differences over 180 days. These findings are not supportive of a disease modifying effect. Thus, the mechanisms suggested should be regarded as speculative—merely hypotheses based upon symptom improvement rather than proof of structural change. Future trials will have to incorporate objective biomarkers (e.g., CTX-II, CPII) or advanced imaging endpoints (e.g., compositional MRI) in order to better evaluate possible effects on cartilage structure (8, 9).

The external validity of this trial is restricted due to a number of design decisions. Although it was methodologically necessary to exclude subjects on concomitant therapies such as NSAIDs, glucosamine or chondroitin to determine the effect of LMCP and to avoid confounding, it creates an experimental setting that is not typical of clinical practice (10, 11) where LMCP would usually be administered with other therapy modalities. This precludes applicability to multimodal OA management. The reported medication adherence of 95–97% to a six-tablet daily regime for 6 months is unreasonably high, and perhaps may not be able to be reproduced in clinical practice. The study's external validity is also limited by the exclusion of patients with BMI ≥30 kg/m^2^, a group with high knee OA prevalence and burden.

In summary, the trial by Park et al. provides early evidence that LMCP may improve symptoms in some people with early knee OA, although the methodological flaws must be taken into consideration. The problems, however, with internal validity (such as randomization imbalance, attrition, analysis set choice), inconsistent outcomes, and unsubstantiated mechanistic hypotheses make it impossible to draw any definitive conclusions about LMCP effectiveness or its place in clinical practice. Future studies should enforce rigorous randomization and allocation concealment, pre-specify intention-to-treat analysis, utilize active comparators (e.g., vs. NSAIDs) and add-on designs to appraise incremental efficacy and report responder analyses. Progressing beyond symptom relief, future studies might also incorporate objective endpoints such as serum biomarkers of cartilage turnover and compositional MRI. Before LMCP can be legitimately considered as a credible option in evidence-based knee OA management, independent replication in larger and more heterogeneous cohorts will be required.
